# Mentorship on malaria microscopy diagnostic service in Ethiopia: baseline competency of microscopists and performance of health facilities

**DOI:** 10.1186/s12936-021-03655-9

**Published:** 2021-02-25

**Authors:** Bokretsion Gidey, Desalegn Nega, Adugna Abera, Abnet Abebe, Sindew Mekasha, Geremew Tasew, Mebrahtom Haile, Dereje Dillu, Degu Mehari, Ashenafi Assefa, Wondimeneh Liknew, Abeba G/Tsadik, Hussien Mohammed, Ermias Woldie, Tsegaye Getachew, Desalegn Ararso, Dereje Yenealem, Adisu Kebede, Kebede Etana, Gizachew Kedida, Hiwot Solomon, Getachew Tollera, Adugna Woyessa, Ebba Abate

**Affiliations:** 1 Ethiopian Public Health Institute (EPHI), Patriot Street, Gulele Subcity, PO Box 1242, Addis Ababa, Ethiopia; 2 Federal Ministry of Health (FMoH), Sudan Street, PO Box 80002, Addis Ababa, Ethiopia; 3 Ethiopian Medical Laboratory Associations (EMLA), Tewodros Square, PO Box: 4866, Addis Ababa, Ethiopia

**Keywords:** External quality assurance, Mentorship, Malaria microscopy, Re-checking, Competency

## Abstract

**Background:**

In Ethiopia, malaria cases are declining as a result of proven interventions, and in 2017 the country launched a malaria elimination strategy in targeted settings. Accurate malaria diagnosis and prompt treatment are the key components of the strategy to prevent morbidity and stop the continuation of transmission. However, the quality of microscopic diagnosis in general is deteriorating as malaria burden declines. This study was carried out to evaluate the competency of microscopists and the performance of health facilities on malaria microscopic diagnosis.

**Methods:**

A cross-sectional study was conducted from 1 August to 30 September, 2019 in 9 regional states and one city administration. A standard checklist was used for on-site evaluation, archived patient slides were re-checked and proficiency of microscopists was tested using a WHO-certified set of slides from the national slide bank at the Ethiopian Public Health Institute (EPHI). The strength of agreement, sensitivity, specificity, and positive and negative predictive values were calculated.

**Results:**

In this study, 102 health facilities (84 health centres and 18 hospitals) were included, from which 202 laboratory professionals participated. In slide re-checking, moderate agreement (agreement (A): 76.0%; Kappa (K)*:* 0.41) was observed between experts and microscopists on malaria detection in all health facilities. The sensitivity and specificity of routine slide reading and the re-checking results were 78.1 and 80.7%, respectively. Likewise, positive predictive value of 65.1% and negative predictive value of 88.8% were scored in the routine diagnosis. By panel testing, a substantial overall agreement (A: 91.8%; K: 0.79) was observed between microscopists and experts in detecting malaria parasites. The sensitivity and specificity in the detection of malaria parasites was 92.7 and 89.1%, respectively. In identifying species, a slight agreement (A: 57%; K: 0.18) was observed between microscopists and experts.

**Conclusion:**

The study found significant false positive and false negative results in routine microscopy on slide re-checking of *Plasmodium* parasites. Moreover, reduced grade in parasite species identification was reported on the panel tests. Implementing comprehensive malaria microscopy mentorship, in-service training and supportive supervision are key strategies to improve the overall performance of health facilities in malaria microscopy.

## Background

Malaria remains a major public health challenge. In 2019, an estimated 229 million cases of malaria and 409,000 deaths were reported worldwide [1] compared with 238 million cases and 736,000 deaths in 2000, which shows a significant decline. In Ethiopia, around 52% of the country’s population is at risk of the disease. Generally, areas that lay below 2000 m above sea level are considered malarious. *Plasmodium falciparum* accounts for nearly 70% of all malaria cases while the remaining cases are due to *Plasmodium vivax,* but malaria prevalence is collectively declining from 2011 (4.5%) to 2015 (1.2%) [2, 3].

The malaria elimination programme in Ethiopia aims to eliminate malaria through a step-wise and sub-national approach targeting specific adjacent areas in order to shrink the country’s malaria map by 2030 [2]. Accurate diagnosis and prompt treatment are core strategies in the elimination of malaria [4]. Diagnosis of malaria is carried out by detecting evidence of parasites or parts of parasites. Microscopic examination of Giemsa-stained blood film is the gold standard for malaria diagnosis [5]. Due to a lack of a sustainable quality assurance programme and trained laboratory technicians, this method has many setbacks in detecting and identifying malaria species correctly in Ethiopia [6–9]. Inaccuracies in diagnostic testing can lead to potentially devastating outcomes for patient and public health, compromising the quality of surveillance data at national level, and ultimately affecting public health policy [10].

Effective malaria diagnosis practice has to be put in place to implement a quality management system that is in line with international quality standards [11]. Strong laboratory capacity with full equipment, reagents and competent professionals ensures better curative interventions and influences treatment-seeking behavior [9] by attracting higher patient flow than facilities with a weak laboratory service. Incorporating a mentoring approach into supervision can transform traditional supervision into a more effective intervention to improve the quality and delivery of patient care [12]. Mentoring typically includes a continued relationship and a broad skills transfer from an individual with more experience in an area to a less experienced mentee in order to improve the performance of laboratory personnel in a health facility [13–16]. An acceptable malaria microscopy service should provide results that are consistently accurate and timely enough to have a direct impact on treatment [15]. This requires comprehensive mentorship and an active quality assurance scheme. This study aimed to evaluate the competency of microscopists and the performance of health facilities on malaria microscopy in Ethiopia. A comprehensive mentorship was provided based on identified gaps.

## Methods

### Study area, design and period

**Fig. 1:**
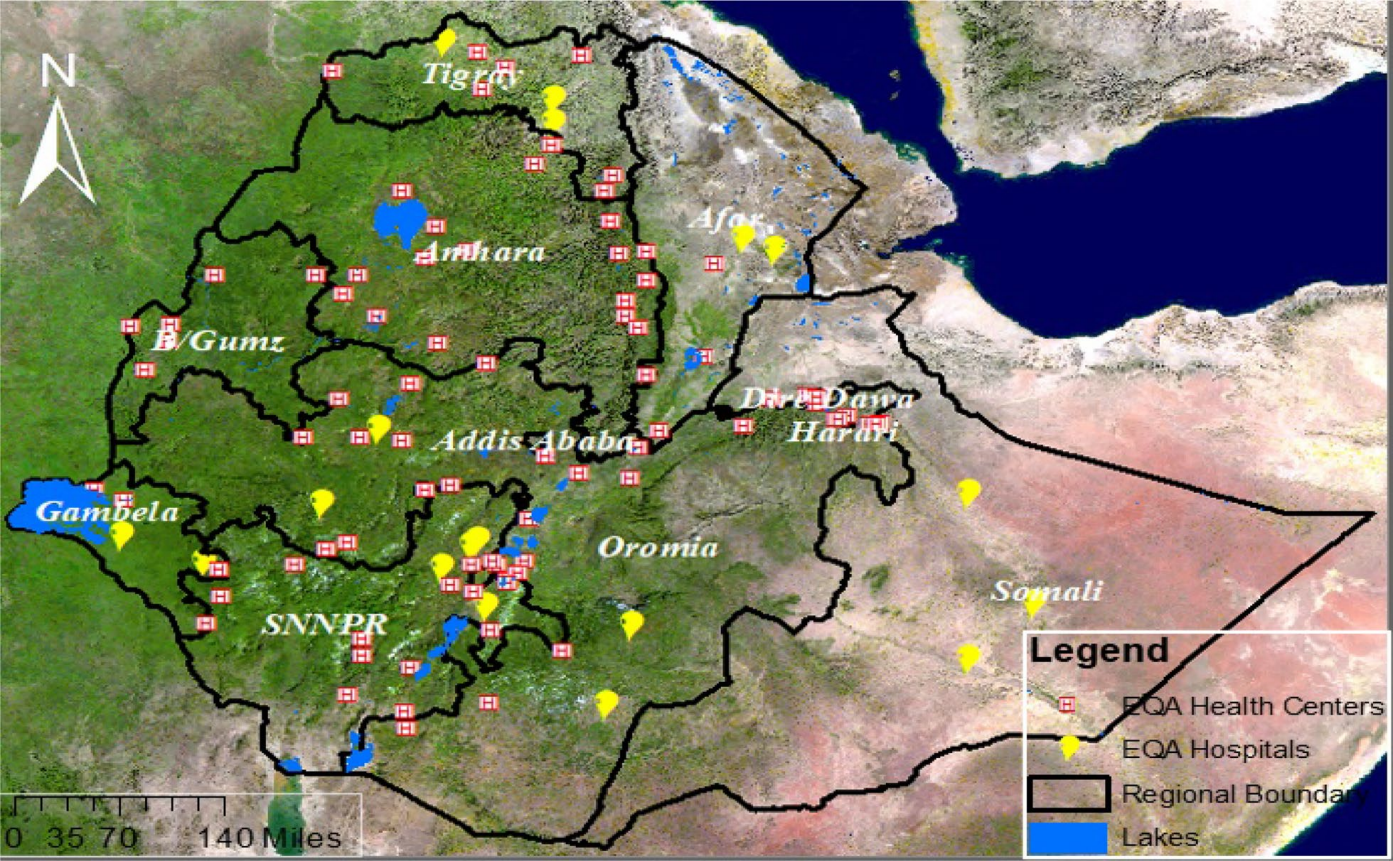
Spatial distribution of participating health facilities (N = 102) in Ethiopia, 2019

A health facility-based, cross-sectional study was conducted from 1 August to 30 September, 2019 at 102 health facility laboratories in 9regional states and one city administration in Ethiopia and the spatial distribution of study sites is depicted in Fig. 1.

### Sample size and selection procedure

In this study, 102 *woredas* (districts) were selected from each region proportionally according to malaria-reporting health facilities based on annual parasite incidence [17]. The mentor team, in coordination with *woreda* health offices, selected one health facility per *woreda* based on their malaria burden report, the presence of medical laboratory professionals and active malaria microscopy diagnostic service.

### Expert selection

Twenty-six experts, 2 from each respective regional Public Health Institute, were selected. Experts were selected based on their experience on malaria microscopy, training on malaria diagnosis and quality assurance, demonstrate ability to transfer knowledge, commitment and willingness to mentor a facility for 3 days with 2 health facilities per week and 8 per month. Five days of standardization training was provided on malaria microscopy and mentorship.

### Data collection process

Thirteen malaria microscopy expert teams, each team with 2 laboratory experts were formed. The experts conducted interviews using a standardized questionnaire using the Open Data Kit (ODK) software programmed on a Smartphone. Panel slides were distributed for proficiency testing. Slides were blind re-checked at health facility level and discordant slides were referred to the national laboratory for confirmation. Real-time data were sent to the Ethiopian Public Health Institute (EPHI) data server immediately after data collection completion at each health facility.

### On-site evaluation

A standard supervisory checklist was used to assess the status of documents and records of quality indicators, quality of smear slides, microscopy diagnosis methods and safety practice, equipment and supply management, capacity building issue and training related to malaria microscopy in each health facility.

### Proficiency testing

A set of 4standard reference slides from the national malaria slide bank at EPHI (one slide *P. falciparum*, one *P. vivax*, one mixed (*P. falciparum* + *P. vivax)* and one negative) were used to test the proficiency of microscopists for parasite detection and species identification.

### Slide re-checking

Expert microscopists (mentors) selected 10stained slides randomly and systematically from the archive of each participant laboratory (5 negative and 5 positive stained slides) as per national external quality assurance (EQA) guidelines [9], and assessed health facilities’ performance in malaria microscopy diagnosis. Slides were read by experts independently at health facility level and the results were compared immediately.

### Data management and statistical analysis

Real-time data were downloaded from the server, cleaned in Microsoft Excel and imported to SPSS V20 for analysis. Descriptive statistics were used to determine quality monitoring indicators, training, performance of health facilities in malaria microscopy, and competence of laboratory professionals. Sensitivity, specificity, percent agreement, and kappa values were used to determine the competency of laboratory personnel against the expert reader in routine diagnosis. Kappa value was calculated to see the strength of agreement. Strength of agreement was classified as: Kappa of < 0.20 is slight agreement, 0.21–0.40 fair agreement, 0.41–0.60 moderate agreement, 0.61–0.80 substantial agreement, and 0.81–0.99 almost perfect agreement [18].

### Ethical consideration

Ethical approval was obtained from the EPHI Institutional Review Board (IRB) (Protocol number: EPHI-IRB-197–2019). An official cooperation letter was written to each health facility. Administrative approvals were obtained from the directors of the participating health facilities.

## Results

### Demographic characteristics

A total of 102 health facilities were enrolled: 18 (17.6%) hospitals and 84 (82.4%) health centres from the 9 regional states and one city administration, in Ethiopia. Two-hundred and two laboratory professionals were included as participants in the competence assessment. The median age of the study participants was 29 years (range: 20–55 years). Most of the participants (68.5%) were male and 3 in 4 participants, 154 (76.2%), reported that they had a diploma educational level in medical laboratory technician. Out of the 202 laboratory personnel, 158 (78.2%) were working at health centres. More than half, 107 (53.0%), of the laboratory personnel had 5 years or more work experience in malaria microscopy (Table 1).

**Table 1:** Demographic characteristics of health facilities and laboratory personnel, Ethiopia, 2019

Characteristics	Variable	Frequency	Percentage (%)
Health facilities enrolled (102)	Hospitals	18	17.6
	Health centres	84	82.4
Age of laboratory personnel (years)	20–30	150	74.2
	31–40	44	21.8
	≥ 41	8	4.0
	Total	202	100
Gender	Male	139	68.8
	Female	63	31.2
Type of college attended	Government	121	59.9
	Private	81	40.1
Education level	Diploma	154	76.2
	BSc	48	23.8
Work Experience (in years)	< 2	38	18.8
	2–5	57	28.2
	> 5	107	53.0
Place of work	Health centre	158	78.2
	Hospital	44	21.8

## Proficiency testing: detection of malaria parasites and species identification

### Overall performance on detection of parasites

A substantial percent agreement (91.8%; Kappa 0.79) was observed between study participants and expert references in detecting malaria parasites. The overall sensitivity of malaria parasite detection by participants using microscopy was 92.7% while the specificity was 89.1%. In the study, 92.7% positive predictive value and 89.1% negative predictive values were observed. Almost perfect agreement (92.87%; Kappa: 0.81) was observed at the health centres and substantial agreement (88.1%; Kappa*:* 0.71) was observed at hospitals in detecting malaria parasites. The overall score for sensitivity of malaria detection in health centres was 94.5% which was higher than in hospitals (86.4%). On the other hand, the specificity of participants in detecting malaria parasites was higher in hospitals (93.2%) than in health centres (87.9%) (Table 2).

**Table 2:** Detection performance of malaria parasites between participants and experts in Ethiopia, 2019

Variables		Expert reading/PT			Agreement (%)	Kappa	PPV (%)	NPV (%)	Sensitivity (%)	Specificity (%)
		Positive	Negative	Total						
Participant	Positive	562	22	584	91.4 (89.18,93.28)	0.79 (0.74–0.83)	96.23 (94.51–97.43)	80.36 (75.40–84.52)	92.7 (90.38–94.68)	89.1 (83.98–93.05)
	Negative	44	180	224						
	Total	606	202	808						
Health centres	Positive	448	19	467	92.87 (90.59–94.76)	0.81 (0.76–0.86)	95.9 (93.92–97.30)	84.24 (78.55–86.64)	94.5 (92.7–96.39)	87.9 (81.86–92.6)
	Negative	26	139	165						
	Total	474	158	632						
Hospitals	Positive	114	3	117	88.06 (82.34–92.46)	0.71 (0.60–0.83)	97.44 (92.71–99.13)	69.49 59.54–77.90)	86.4 (79.31–91.71)	93.2 (81.34–98.57)
	Negative	18	41	59						
	Total	132	44	176						

*PPV* Positive predictive value, *NPV* Negative predictive value, *PT* Proficiency testing

### Overall percent agreement on species identification

Slight agreement (57%; Kappa: 0.18) was observed between participants and expert readers in identifying *P. falciparum* from non-*P. falciparum* parasites. The overall percent agreement in identifying *P. falciparum* and non*-P. falciparum* was 49% (Kappa: 0.04) at hospital level and 59% (Kappa: 0.22) at health centre level. The percent agreement in species identification at health centres was slightly higher than agreement at the hospitals despite low overall agreement at all facilities (Table 3).

**Table 3:** Identification performance of *Plasmodium falciparum* against non-*falciparum* parasites in Ethiopia, 2019

Variables		Expert reading			Agreement (%)	Kappa
		*P. falciparum*	Non*-P. falciparum*	Total		
Participants’ results	*P. falciparum*	147	205	352	57 (53.05–61.08)	0.18 (0.11–0.26)
	Non*- P. falciparum*	55	199	254		
	Total	202	404	606		
Health centre	*P. falciparum*	119	154	273	59 (54.71–63.74)	0.22 (0.15–0.30)
	Non- *P. falciparum*	39	162	201		
	Total	158	316	474		
Hospital	*P. falciparum*	28	51	79	49 (40.44–58.08)	0.04 (− 0.1 to 0.19)
	Non- *P. falciparum*	16	37	53		
	Total	44	88	132		

## Performance of health facilities in malaria microscopy diagnosis

### Slide re-checking

Seven-hundred and fifty blood film slides (maximum 10 blood film slides) from each of the 102 assessed health facilities were re-checked by experts. Compared with expert readers, percent agreement of the experts and facilities resulted in malaria parasite detection 76.0% (Kappa*:* 0.41) which was at the base boundary of moderate agreement. The sensitivity and specificity were 78.1 and 80.7%, respectively. Similarly, positive and negative predictive values were 65.1 and 88.8%, respectively (Table 4).

**Table 4:** Detection of the malaria parasites in slide re-checking, Ethiopia, 2019

Slides from health facilities		Expert reading			Agreement	Kappa	Sensitivity	Specificity	PPV	NPV
		Positive	Negative	Total						
Health facilities’ results	Positive	185	99	284	76.0%	0.41	78.1%	80.7%	65.1%	88.8%
	Negative	52	414	466						
	Total	237	513	750						

### On-site evaluation

Only 72 (70.5%) of the health facilities were performing thick and thin smears on a single slide for every patient. Three in 10 health facilities did not use thin smear for species identification. Less than half the health facilities, 46 (45.1%), monitor the quality of prepared blood film slides regularly. Only one in 3 health facilities, 38 (37.3%), perform regular internal quality control (IQC) of Giemsa solution and more than 2 in 3 health facilities, 65 (64.7%), did not store or archive examined slides properly. As part of quality assurance, one of the observed major challenges in most facilities was failure to archive and store examined slides properly, except in 37 (37.3%) of the health facilities, and only half of 51 (50%) of the health facilities were participating regularly in any method of external quality assurance (Table 5). Sixty-one (59.8%) health facilities had been supervised by national and/or regional health offices in the preceding year. Regular training on malaria diagnosis and quality assurance was reported at 26 (25.5%) health facilities. Thirty-two (31.4%) health facilities diagnosed malaria using both rapid diagnostic tests and microscopy (Table 5).

**Table 5:** Assessment of quality assurance indicators on malaria microscopy, Ethiopia, 2019

Assessment questions	Response	Frequency	Percent (%)
Malaria diagnosis method used	Microscopy only	68	66.7
	RDTs only	2	1.9
	Both	32	31.4
Prepare both thick and thin films on one slide	Yes	72	70.5
	No	30	29.5
Monitor the quality of prepared blood film slides	Yes	46	45.1
	No	56	54.9
Routinely perform IQC in a regular manner	Yes	38	37.3
	No	64	62.7
Report malaria density or counting malaria parasites in a routine examination	1^+^, 2^+^, 3^+^,4^+^	31	30.4
	Parasite/µl	0	0
	Infected RBCs (%)	0	0
Store and archive examined slides properly	Yes	37	36.3
	No	65	64.7
Supervised by regional and/or national levels in the last year	Yes	61	59.8
	No	41	40.2
Obtain refresher training on malaria microscopy diagnosis	Yes	26	25.5
	No	76	74.5
Participate in any EQA programme regularly	Yes	51	50
	No	51	50
Quality of prepared and archived slides in the health facilities	Excellent	8	7.8
	Good	51	50
	Poor	43	42.2

**Box 1:** Criteria for assessing quality of malaria blood film slide according to national malaria laboratory diagnosis EQA scheme guideline [9]

Grading	Criteria
Excellent	Gross appearance: Both thin and thick film prepared on the same slide, thick film 10 mm diameter, newsprint read under thick film before staining, 10 mm from frosted end and thick film and between thick and a thin film with distinct head, body and tail Microscopic appearances: Demonstrates RBCs lysed in thick film and a monolayer of RBCs, with normal and abnormal morphology in thin film. Staining allows the trophozoites, gametocytes and/or schizonts and the white blood cells to be clearly distinguished against the background
Good	Gross appearance: Film with uneven tail, too thick, too wide or too long with uneven thickness Microscopic appearance: Demonstrates a monolayer of RBCs, and fixed RBCs Staining allows the trophozoites, gametocytes and/or schizonts malaria parasites and the white blood cells to be clearly distinguished against the background
Poor	Gross appearance: Film with ragged tail, too thick, too wide or too long with uneven thickness Microscopic appearance: Distorted appearance of the RBCs, malaria parasite and the white cells. It is difficult to spot fields with monolayer of cells and distorted appearance of the RBCs, malaria parasite, and the white cells

## Discussion

In routine microscopic diagnosis, moderate performance agreement (A) (A: 76.0%; Kappa: 0.41%) was observed between study facilities and expert microscopists in parasite detection at all the health facilities. This study showed lower performance agreement compared to a study from the West Amhara Region of Ethiopia [19, 20] and Hawassa, southern Ethiopia [21]. The discrepancy may be due to the large number of health facilities used in the current study. Sensitivity and specificity at detecting malaria in peripheral blood-stained slides were 78.1 and 80.7%, respectively. Similarly, the positive and negative predictive values were 65.1 and 88.8%, respectively. High false positive and false negative results were found at the assessed health facilities, which showed poor performance of their routine diagnosis of malaria. This result was lower than the findings reported from other parts of Ethiopia [19, 20, 22], and similar findings were reported in Pakistan [23]. However, the findings of this study were in line with a study from the Democratic Republic of Congo, which showed performance of routine malaria microscopy remained inaccurate, with large variations among different health centres [24]. Accurate microscopy results depend on the availability of a competent microscopists using good-quality reagents for examining well-prepared slides and with a low-to-moderate workload [25]. In this finding, in slide re-checking, almost half of the assessed health facility laboratories did not prepare both thick and thin blood films as per standard. Consequently, low sensitivity in detection of malaria parasites indicated that there were many false-negative results, i.e., missed diagnosis of true infection. This can lead to delayed treatment, development of serious complications, and death or exposure to unnecessary treatment with other drugs due to suspecting other fever-like diseases.

Regarding proficiency testing, the overall percent agreement of the malaria microscopists in the current study was 91.8% (Kappa: 0.79) in parasite detection, which is relatively high when compared with similar findings at elimination-targeted districts of Ethiopia where performance agreement was 84.6% (Kappa: 0.6) [26]. A study conducted in Hawassa town, Ethiopia, reported an agreement of 88% (Kappa: 0.67) [21] and similar findings were reported from a study conducted in Bahirdar, Ethiopia where agreement was 88.5% (Kappa: 0.78) [27]. A study conducted in Tigray, Ethiopia, reported an agreement of 79% (Kappa*:* 0.62) [28]. A concordant result reported from Addis Ababa public health facilities showed a performance agreement of 91.7% [29]. But the agreement of the current study was relatively low when compared with findings of similar studies conducted in Ethiopia where the agreement was 96.8% (Kappa: 0.9) [28]. The reason for this deviation may be because of the difference in malaria prevalence which can affect a microscopist’s ability to detect, but could also be the lack of mentorship, training, consistent supervision, and capacity building used to develop detecting skills and standardize malaria parasite detection. Overall, sensitivity and specificity of laboratory personnel in detecting malaria parasites were 92.7 and 89.1%, respectively. These results overlapped with positive predictive value (92.7%) and negative predictive value (89.1%). These findings were almost in agreement with a sensitivity 88% and specificity 91% from a study conducted in Zambia [30]. The sensitivity but not the specificity of this study was higher than the sensitivity 83.2% and specificity 90.1% in a study conducted elsewhere in Ethiopia [26], and sensitivity 63% and specificity 97% in a study reported in Tigray, Ethiopia [28]. The sensitivity and specificity of this study was lower than the sensitivity 96.8% and specificity 96.7% of malaria detection in a study conducted in Tigray, Ethiopia [28]. The relative lower specificity than sensitivity in the current study at detecting malaria parasites showed that a high rate of false positive results were reported, which led to misdiagnosis of malaria when there was no true infection of malaria parasite in the provided slides.

Overall, the performance agreement on identification of malaria species was 57% (Kappa: 0.18), which showed a slight agreement between participants and malaria microscopy experts. This result was higher than in a similar study conducted in elimination-targeted districts in Ethiopia with an agreement of 43.8% (Kappa*:* 0.11) [26] while it was lower than in a study conducted in Tigray, Ethiopia with an agreement of 76% (Kappa: 0.61) [28] and a study reported from Bahirdar, Ethiopia where the agreement was 72% (Kappa: 0.47) [27]. The reason for the low identification of species by microscopists in the current study may be due to the microscopists preparing a thick film only and being unable to differentiate the morphology of the parasites. It may also be due to the lack of training on how to differentiate the species, and to poor staining of reagents found at on-site evaluation.

At on-site evaluation, only 72(70.5%) health facilities were performing thick and thin smears on a single slide for every patient, but the recommended blood film preparation for diagnosis of malaria parasites is both thin and thick films on the same slide using 2µL and 6µL of whole blood, respectively [31]. Three in 10 health facilities were not using thin smears, which can be used for species identification. This result in the current study is greater than a study conducted in Addis Ababa public health facilities [29]. Moreover, blood films performed in 43 (42.2%) health facilities did not meet the quality of a good blood film for malaria microscopy diagnosis in this study. IQC, used to check the quality of Giemsa stains, was performed only by 38(37.3%) health facilities. Only 37(37.3%) of health facilities stored and archived slides properly. Moreover, in the previous year, the study identified in 41 (40.2%) and 76 (74.5%) health facilities with no supervision and refresher training, respectively. This study was in line with a study conducted in Addis Ababa public health facilities [29] and another study conducted in Asia–Pacific [32]. The results of this study showed that less attention is given to the quality of malaria diagnosis at health facility level. It may be due to lack of refresher training and regular supervision provided to laboratory professionals at health facility level.

## Limitations

There were some limitations where the study health facilities were incorporated purposely based on their high malaria load and presence of microscopic service and the findings are not inferred to occur at all health facilities in the country. The health facilities with no or lower malaria reporting were not included. In addition, a low numbers of slides (3 malaria positive and one negative slide) were used in panel testing, which is below WHO standard of 10 slides.

## Conclusion

Most of the malaria microscopists in the current study achieved a good grade agreement in parasite detection. However, a poor grade was obtained in parasite species identification by the panel tests. Moreover, high false positive and false negative results were seen on slide re-checking which showed poor performance of the health facilities in routine malaria microscopy. Poor quality control indicators and follow-up gaps were reported by a significant number of health facilities in this study. An improvement in the quality and accuracy of microscopic diagnosis of malaria is urgently needed. A strong commitment from the National Malaria Elimination Programme and from stakeholders is the vital step to accomplish a mentoring approach at all levels of health facilities.

## Data Availability

The dataset and materials used for the study are kept in a safe place on the EPHI server.

## Abbreviations

EPHI: Ethiopian Public Health Institute
EQA: External quality assurance
EQAS: External quality assessment
NMEP: National Malaria Elimination Programme
GPS: Global positioning system
IQC: Internal quality control
IRB: Institutional review board
ODK: Open data kit
PT: Proficiency/panel testing
RDTs: Rapid diagnostic tests
SERO: Scientific and ethical review office
PPV: Positive predictive value
NPV: Negative predictive value
